# Supplementary material for: An Amplicon-Based Approach for the Whole-Genome Sequencing of Human Metapneumovirus
Source: Viruses. 2021 Mar 18;13(3):499. doi: 10.3390/v13030499 (PMC8003040; doi:10.3390/v13030499)
Supplement: Supplementary file 1 [file viruses-13-00499-s001.zip › Sup Fig 2.pdf]

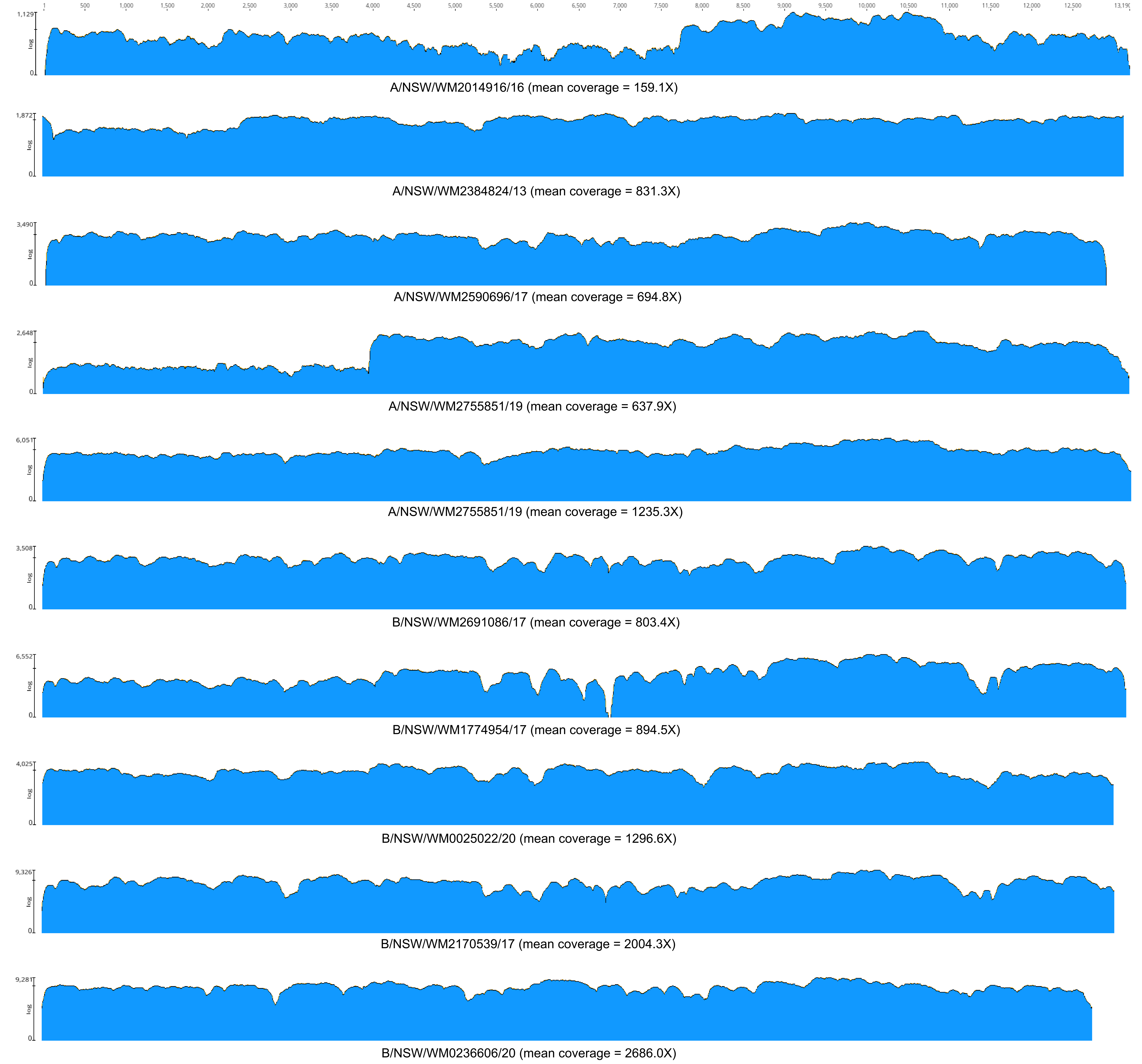

Supplementary Figure 2. Coverage plots for the ten HMPV whole genome sequences generated in this study. The trimmed sequence reads were mapped onto draft reference genomes to examine coverage along the virus genome. The y-axis shows the log-scaled sequence reads depth (coverage) and the x-axis shows the relative genome position according to the scale at the top. The mean coverage is provided for each sample.
