# Supplementary figures and images for: An Amplicon-Based Approach for the Whole-Genome Sequencing of Human Metapneumovirus
Source: Viruses. 2021 Mar 18;13(3):499. doi: 10.3390/v13030499 (PMC8003040; doi:10.3390/v13030499)

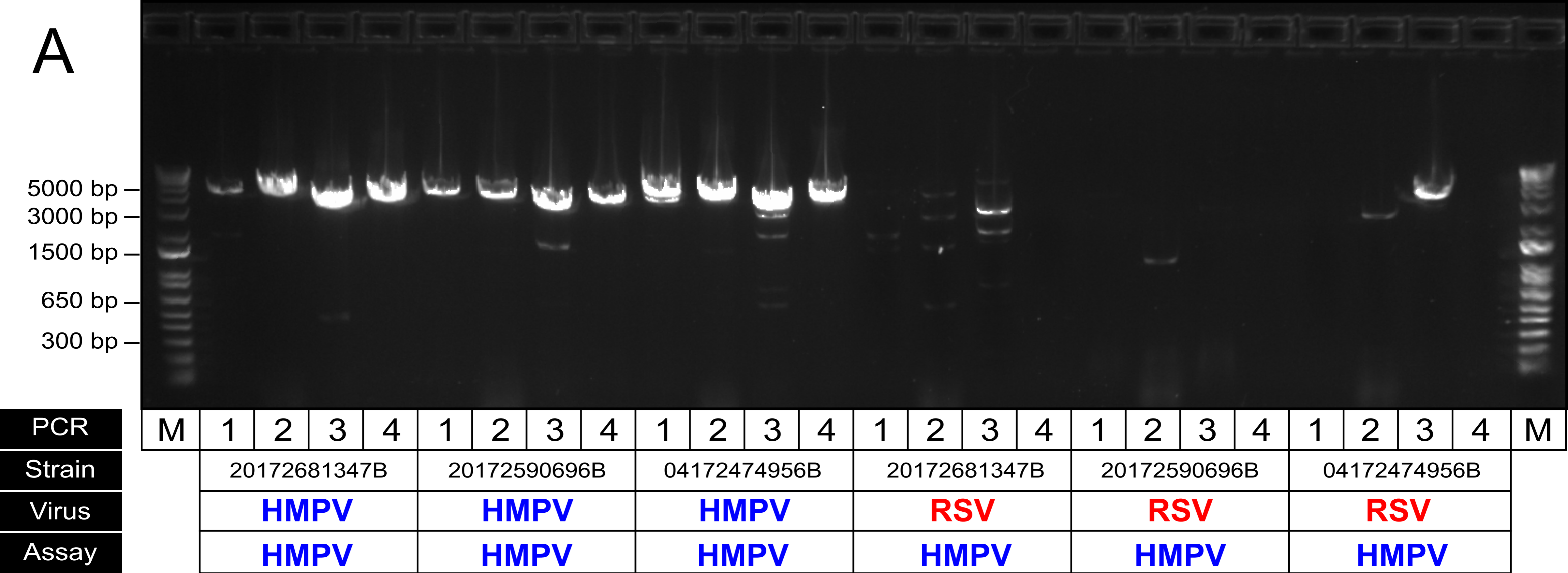

Supplement: Supplementary file 1 [file viruses-13-00499-s001.zip › Sup Fig 1.pdf]
